# Pyelonephritis in Pregnancy From the Lens of an Underserved Community

**DOI:** 10.7759/cureus.29029

**Published:** 2022-09-11

**Authors:** Chioma C Umeh, Okelue E Okobi, Olamide I Olawoye, Chukwuebuka Agu, Jovita Koko, Joseph Okoeguale

**Affiliations:** 1 Family Medicine, Sure Hope Hospital, Lagos, NGA; 2 Family Medicine, Lakeside Medical Center, Belle Glade, USA; 3 Internal Medicine, Uniosun Teaching Hospital, Osun, NGA; 4 Family Medicine, Parkland Integrated Health Center, Shellbrook, CAN; 5 Medicine and Surgery, University of Jos, Jos, NGA; 6 Obstetrics and Gynaecology, Irrua Specialist Teaching Hospital, Irrua, NGA

**Keywords:** urinary track infection, complicated uti, high-risk pregnancy, underserved populations, complicated pyelonephritis, pyelonephritis in pregnancy

## Abstract

In pregnancy, early signs and symptoms of urinary tract infection, including cystitis or pyelonephritis, may overlap with pregnancy symptoms, making early detection challenging. Compounding this challenge is when it presents itself in resource-poor settings for several factors, including poverty, poor access to healthcare care, inadequate diagnostic facilities, low availability of insurance, education, and cultural limitations. In this case report, we present a case of a 33-year-old G3P2 with pyelonephritis in pregnancy that was compounded by issues related to access to care in resource-limited settings. Although this case was handled in a resource-poor country, fighting to improve access to better health care, the term "underserved" is not exclusive to such a place. Therefore, we reviewed some basic guidelines for managing pyelonephritis in pregnancy and the obstacles in most underprivileged populations.

## Introduction

Worldwide, pyelonephritis is one of the common presentations of infection in pregnancy [1]. It affects about 1-2% of pregnant women [2] and is a common cause of hospitalization during pregnancy. Pyelonephritis occurs mostly in the second and third trimesters, with about 10-20% occurring in the first trimester [3]. Pregnancy predisposes women to an increased risk of pyelonephritis. The high levels of progesterone cause smooth muscle relaxation and reduced peristalsis in the renal collecting system. The decreased detrusor tone of the bladder leads to incomplete emptying and increased capacity. In addition, the pressure effect of the gravid uterus on the renal system predisposes to various levels of renal calyceal dilatation, leading to stasis of urine and nidus for bacteria to populate themselves. This is further enhanced by the physiological changes in pregnancy of increased levels of proteinuria and glycosuria, encouraging the growth of microorganisms [4]. Other risk factors include sickle cell disease, diabetes mellitus, anemia, structural diseases of the kidney, previous history of urinary tract infection (UTI), sexual activity, and low socio-economic status [5,6]. 

Managing infections during pregnancy in resource-poor settings can be challenging for a number of factors, ranging from poverty, poor access to health care, poor diagnostic facilities, low availability of insurance, education and cultural limitations, etc. In some resource-poor settings, there is poor access to microbiological diagnostic facilities like urine culture and even dipsticks to detect asymptomatic bacteriuria, which eventually affects early diagnosis and prompt treatment [2,7]. They also lack information that guides antibiotic usage [2-4,8]. It is recommended by the Canadian Task Force on Preventive Care [9,10], the Infectious Diseases Society of America [11], and the National Institute of Health and Clinical Excellence of the United Kingdom [12] that all women should be screened and treated for asymptomatic bacteriuria at least once in early pregnancy [13]. This case report discusses a case of pyelonephritis in pregnancy that was compounded by issues related to access to care in resource-limited settings. Despite the fact that this index case was handled in a resource-poor country fighting to improve access to better health care, the term "underserved" is not exclusive to such a place. This report examines the basic guidelines of pyelonephritis in pregnancy and the obstacles in the majority of underprivileged populations, but each underserved location is distinct.

## Case presentation

A 33-year-old G3P2 female with two living children presented at 36 weeks of gestation to the emergency department of a private hospital because of a two-day history of fever, right flank pain, dysuria, loss of appetite, and malaise. She has been on acetaminophen, which temporarily relieved her symptoms. Her past medical and family history has been uneventful. The patient's first antenatal visit was at 34 weeks of gestation, and the details of the visit were apparently normal, including booking urinalysis. Her last confinement was six years ago, which ended in a Cesarean section due to placenta previa. Vital signs on presentation were: temperature of 38.4°^ ^C, heart rate of 113 bpm, blood pressure of 90/50 mmHg, and oxygen saturation (SPO_2_) of 96%. The chest was clinically clear. Abdominal examination demonstrated a gravid uterus of 36 weeks size with obvious fetal movement. There was a marked right loin tenderness. There was no demonstrable tenderness in the other areas of the abdomen. She had no palpable uterine contraction. The fetal heart sound was heard; it was regular 135 bpm using the handheld doppler. Pertinent laboratory findings are presented in Table 1.

**Table 1:** Laboratory findings

Tests	Unit	Result	Normal range
Complete blood count			
Packet cell volume (PCV)	g/dl	29	35 - 49
Hemoglobin (Hb)	%	9.8	12.5 - 15.5
Red blood cell count	10^12^/L	3.3	4.0 - 5.4
Platelet	10^9^/L	440	150 - 450
Total white cell count	10^9^/L	13.1	4.5 - 11.5
Differentials in %			
Neutrophils		72	40 - 70
Lymphocytes		27	20 - 50
Monocytes		1	2 - 10
Eosinophils		0	1 - 6
Basophils		0	0 - 1
Urinalysis			
Appearance		Cloudy	Clear
Color		Yellow	Amber
pH		7.3	4.5 - 8
Protein		Negative	Negative
Glucose		Negative	Negative
Ketones		Negative	Negative
Blood		Negative	Negative
Bilirubin		Negative	Negative
Urobilinogen		Negative	Negative
Specific gravity		1.015	1.005 - 1.030
Leucocytes		+	Absent
Nitrites		++	Absent

**Figure 1:**
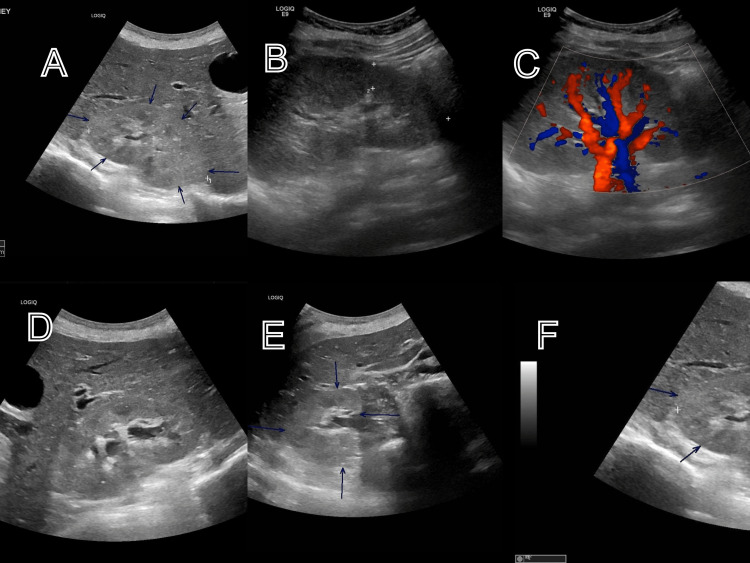
Renal ultrasound with arrows showing some features of pyelonephritis A: loss of corticomedullary differentiation, mixed echogenicity with mild enlargement; B: renal enlargement; C: increased renal perfusion on Doppler; D: modest caliceal dilatation (hydronephrosis); E: transverse plane showing accentuated cortical echogenicity; F: comparison of the echogenicity of the liver and kidneys and kidney outline loss from edema.

The complete metabolic panel, urine culture, and admitting ultrasound scan (US) were declined due to the financial burden on the patient. She was admitted for management of acute pyelonephritis and was started empirically on intravenous amoxicillin potentiated clavulanate (Augmentin®) at 1.2 g eight-hourly for 48 hours and thereafter oral Augmentin® for two weeks. The patient was commenced on intravenous fluids and antipyretics. She was closely monitored for possible deterioration in vital signs. At about 18 hours post admission, fetal tachycardia was noted. Fetal heart rate (FHR) fluctuated between 165 and 185 bpm, with no palpable contraction. She was still febrile with an increasing temperature of 39°​​​​​​​ C, a heart rate of 120 bpm, and a blood pressure of 90/50 mmHg. The renal US and US for fetal monitoring/biophysical profile were done at this point, showing some features typical for pyelonephritis (Figure 1). 

The patient subsequently underwent an emergency Cesarean section on account of fetal distress. The APGAR score was six (at one minute), seven (at five minutes), and nine (at ten minutes) in a female with a 2.3 kg birth weight. She did well postoperatively. The patient was fever free on day three of admission and was subsequently discharged home with oral completion of antibiotics to be seen during her post-natal visit.

## Discussion

The problems of delivering high-quality health care and the dangers of failing to satisfy this expectation are not new. In navigating the therapy recommendations for pyelonephritis in pregnancy, this discussion will concentrate on these difficulties, with the aim of proffering a possible solution. First, pyelonephritis in pregnancy has been described in many academic publications as a frequent cause of antepartum hospitalization [1-7]. The disease is an infection or inflammation of the upper urinary tract system. Several potentially overlapping risk factors are thought to be involved in the genesis of this illness, such as frequency of sexual intercourse, medical diseases such as diabetes, sickle cell disease or trait, and neurological issues such as paralysis from spinal cord damage, and instrumentation are examples and low socio-economic status. Pyelonephritis in pregnancy is more likely to occur in people with low socio-economic status [2-4], like the patient in this case report. With incomes universally lower in rural areas, by connotation, rural dwellers, especially those living in a resource-poor setting [14-15], may be seen to bear more of the burden of this disease. This discussion adopted the definition of resource-poor settings as areas with limited means or supply of resources (both human and material) for healthcare [15]. Such settings are also mainly populated by people living below the poverty line and people with low socio-economic status. Although higher percentages of resource-poor settings are seen in developing countries, they are also found in developed nations, predominantly in rural areas; just like this patient, a preponderance of patients with this disease condition present during the second to the third trimester. An observational study by Artero et al. on 93 cases in 2013 found about 88% of study participants presented during the mid to last trimester [16]. However, the late onset of the disease amplifies its complications in rural settings because of the trend where many women living in these resource-poor settings do not register for antenatal care, and even when they do, they register late. Late registration for antenatal care has been a predictor of poor outcomes in pregnancy [2-4, 17], a challenge commonly seen by family physicians and obstetricians who practice in resource-poor settings.

Some early signs and symptoms of urinary tract infection, including those of cystitis or pyelonephritis, may overlap with some symptoms of pregnancy, making early detection challenging. Urinary symptoms, fevers, fatigue, malaise, chills, flank pain, nausea, vomiting, and cystitis symptoms such as dysuria and increased frequency are all early signs of this disorder and may be mistaken as pregnant symptoms in naive patients or unskilled caretakers [1-8]. Strengthening the rural health workforce by continuous education to improve health outcomes in rural communities is a method that several nations have embraced and has been shown to be beneficial [18].

The laboratory diagnostic challenge is another dimension in managing this disease, especially in health facilities in the socio-economic population regions. The syndromic approach model using empirical treatment based on the knowledge of commonly implicated organisms is sometimes helpful in resource-poor settings. Positive urine dipstick in pregnancy is frequently followed by urine culture in various national obstetric or related guidelines across the world [6-8,10-13,16-21]. Although urinary dipstick testing for nitrate and leukocyte esterase may suffice in combination with a physical examination as a diagnostic tool in this condition, in the absence of universal health insurance and coverage, patients may not afford other standard or supporting diagnostic tests, like urine culture and renal ultrasound, that may help improve management outcomes. Many end up declining recommended tests because of affordability. Affordability and accessibility of limited microbiology services hamper diagnosing and treating asymptomatic bacteriuria and UTIs in pregnancy in resource-poor settings [2,19]. In these settings, antenatal care facilities cannot conduct regular urine cultures to identify asymptomatic bacteriuria. They are compelled to depend on dipstick urinalysis as a less expensive alternative, with the limitation of being less sensitive and specific. This leads to an increase in the number of pregnant women with upper urinary tract infections and the complications that come with them. It is estimated that between 1.8 and 30% of pregnant women with untreated asymptomatic bacteriuria will develop pyelonephritis [2-4]. This was the situation with this patient, who presented with a urinalysis and no culture. However, urinalysis may not be relied on since dipstick has a sensitivity and specificity of about 38.7 percent and 35.8 percent, respectively [2-9], resulting in missed asymptomatic bacteriuria. Urine microscopy, culture, and sensitivity are superior options and are recommended to be included in urine analysis [10-13]. It has a sensitivity and specificity of 71% and 73.6%, respectively [2,5-9]. Urinary tract infection is defined as positive urine for bacteriuria, leukocyte esterase, nitrite, >6-12 white blood cells, and >2-4 epithelial cells, among other things. The sensitivity and specificities of these numerous parameters vary [20]. Diagnosis is a combination of at least one of the systemic symptoms like fever (>38° C), flank pain, or urine culture with >10^5^ colony-forming units [7-9,16,21].

The role of radiological imaging has been explored as well. In pregnancy, renal ultrasonography is the preferred imaging technique over CT imaging to limit the danger of radiation exposure to the fetus unless when absolutely indicated. The typical ultrasound findings may include enlargement of the affected kidney; there may be segmental abnormal echotexture in the affected area, more hypoechogenicity, varying degree of renal blood flow (increased and decreased) to the inflamed area on doppler evaluation, echoes in the affected segmental collecting duct, features of hydronephrosis, loss of cortical hepato-renal differentiation, and mass-like appearance of affected segments, possibly causing mass-effect on the normal adjacent parenchyma.

Once a clinical suspicion is made, and a laboratory diagnosis is confirmed, prompt treatment with appropriate intravenous antimicrobials is usually instituted. The evidential support treatments are usually focused on the elimination of the usual uropathogenic causative organisms (mostly gram negatives and group B Streptococcus); it remains unclear between outpatient intravenous administration and inpatient [9-13,21]. However, an inpatient approach is favored because of the additional benefit of fetal monitoring. Several antibiotic regimens may be used; however, recent evidence evaluating three regimens shows no difference in hospitalization time, preterm delivery, or premature birth [21,22]. 

Complications of pyelonephritis can be both maternal and fetal. They include preterm labor, preterm birth, intrauterine growth restriction, acute respiratory distress syndrome, sepsis, acute kidney injury, and maternal or fetal death. Unfortunately, preterm labor and low birth weight was the outcome of this case. According to some studies, about 20 to 40% of pregnant women with pyelonephritis have preterm births, and these infants are at the highest risk of neonatal death in these resource-poor settings [2,19,21]. A study estimated that screening and early treatment of bacteriuria in pregnancy might lower the incidence of preterm deliveries and low birth weight by 20% to 55% and reduce associated neonatal mortality by 5% to 14% [19,21]. Treatment failure presenting as persistent fever despite adequate therapy may occur due to antimicrobial resistance, abscess, urolithiasis, or structural anomalies. 

Another difficulty in delivering quality health care in resource-poor settings is disease prevention. One of the approaches to disease prevention by primary care providers is using disease prevention methods. Asymptomatic bacteriuria carries an increased risk of pyelonephritis in pregnancy [16]. Some studies have shown that with routine screening for asymptomatic bacteriuria and consequent aggressive treatment, it is possible to reduce the incidence of urinary tract infections and their complications during pregnancy [19,21]. However, the debate continues to reverberate about its cost-benefit effects. According to the Canadian Agency for Drugs and Technologies, one randomized study and four evidence-based guidelines were identified regarding routine urinalysis for low-risk pregnancies. Two of the four guidelines discovered were written by National Institute for Health and Care Excellence (NICE), one was released by the Society of Obstetricians and Gynecologists of Canada (SOGC), and one was published by the Institute for Clinical Systems Improvement (ICSI). Two guidelines advise against using urinalysis to screen for certain illnesses in pregnant women, while two others recommend it as a regular screening test [23]. 

Other hurdles in early screening of UTIs in pregnancy in resource-limited settings include inadequacy in the availability of antenatal services, high illiteracy rates, poor health-seeking behaviors, and contaminated urine samples due to poor education on collection methods and, of course, a lack of political will and disproportionately low resource allocation, including the lack of national health insurance coverage for the population and an equitable allocation of resources. In some resource-poor settings, there is no effective regulation of access to antibiotics, so patients can purchase antibiotics without a prescription, which could result in antibiotic resistance. This case report depicts the fact that screening for asymptomatic bacteriuria and early treatment of UTIs is a cost-efficient way of improving maternal and child health. In the index patient, the underutilization of this useful screening tool during the course of the pregnancy and the associated diagnostic urine culture could have contributed to the poor outcome of the pregnancy. The potential of utilizing disease prevention strategies in underserved regions can be reinforced by focused health care interventions like universal health insurance. But unfortunately, the uneven rural-urban distribution of health resources and physicians has been the center of debate in most health systems around the world. 

## Conclusions

One of the most prevalent causes of maternal sepsis is pyelonephritis. This is particularly common in resource-limited situations owing to limited access to care, insurance availability, education, and cultural constraints. Furthermore, there is an overlap between pregnant symptoms and an acute infection, which is often overlooked, leading to consequences. Prompt diagnosis and treatment have the potential to avert maternal and fetal problems. The different limiting constraints in the underserved population, such as a lack of sufficient testing in resource-poor locations and a lack of follow-up or prenatal visits, may worsen the burden of this illness. We recommend that pregnant women and primary care professionals in these places continue to be educated and informed about the disease's preventive and treatment guidelines.
